# Validation of the Updated “LIfestyle for BRAin health” (LIBRA) Index in the English Longitudinal Study of Ageing and Maastricht Aging Study

**DOI:** 10.3233/JAD-240666

**Published:** 2024-10-08

**Authors:** Colin Rosenau, Sebastian Köhler, Martin van Boxtel, Huibert Tange, Kay Deckers

**Affiliations:** a Alzheimer Centrum Limburg, Department of Psychiatry and Neuropsychology, Mental Health and Neuroscience Research Institute (MHeNs), Maastricht University, Maastricht, The Netherlands; b Department of Family Medicine, Care and Public Health Research Institute (CAPHRI), Faculty of Health, Medicine and Life Sciences, Maastricht University, Maastricht, The Netherlands

**Keywords:** Alzheimer’s disease, cognitive dysfunction, dementia, healthy lifestyle, primary prevention, protective factors, risk factors, risk reduction behavior

## Abstract

**Background:**

The “LIfestyle for BRAin health” (LIBRA) index was recently updated with three new modifiable factors: hearing impairment, social contact, and sleep (LIBRA2), but has not yet been validated.

**Objective:**

Comparison of the performance of both LIBRA versions in predicting dementia risk.

**Methods:**

Longitudinal data from the English Longitudinal Study of Ageing (ELSA) and the Maastricht Aging Study (MAAS) were used. The weighted LIBRA (11/12 factors available) and LIBRA2 (14/15 factors available) scores were calculated, with higher scores representing an unhealthier lifestyle. Dementia diagnoses were based on self- or informant reported physician diagnosis, an informant-based cognitive screening tool, registry data or test data. Cox-proportional hazards regression was used to investigate the association between LIBRA(2) scores and dementia risk. Model fit and predictive accuracy were determined using the Akaike information criterion and Harrell’s C index.

**Results:**

Over an average follow-up of 8.3 years in ELSA and 17.9 years in MAAS, 346 (4.6%) and 120 (8.5%) individuals developed dementia, respectively. In ELSA, a one-point increase in LIBRA2 was associated with an 8% (1.06–1.11) higher dementia risk (LIBRA: 13%, 1.09–1.16). In MAAS, a one-point increase in LIBRA2 was associated with a 6% (1.01–1.12) higher dementia risk (LIBRA: 8%, 0.99–1.16). In ELSA, LIBRA (Harrell’s C = 0.68) and LIBRA2 (Harrell’s C = 0.67) performed similarly. In MAAS, LIBRA2 (Harrell’s C = 0.62) performed better compared to LIBRA (Harrell’s C = 0.52)

**Conclusions:**

LIBRA2 is a better model for identifying individuals at increased dementia risk and for public health initiatives aimed at dementia risk reduction.

## INTRODUCTION

The number of people living with dementia is estimated to increase from 57 million in 2019 to 153 million in 2050, prompting the World Health Organization to declare dementia a global health priority and emphasizing the importance of developing validated risk scores to identify individuals at increased risk of dementia.^1–3^

In 2013, the “LIfestyle for BRAin health” (LIBRA) index was developed by means of a systematic literature review and Delphi expert consensus study to identify individuals with an increased risk of dementia, transfer knowledge about and increase motivation for lifestyle changes for healthy brain aging and dementia risk reduction. LIBRA exclusively includes modifiable risk and protective factors for cognitive decline and dementia, thereby capturing lifestyle-based prevention potential.^4^ In contrast, other dementia risk scores combined modifiable factors with non-modifiable factors, such as age, sex, and genetics.^5,6^ The original version of the LIBRA index consisted of three protective factors (low-to-moderate alcohol consumption, healthy diet, and high cognitive activity) and nine risk factors (coronary heart disease, physical inactivity, chronic kidney disease, diabetes, midlife hypercholesterolemia, smoking, midlife obesity, midlife hypertension, and depression).

LIBRA has shown to predict cognitive functioning/decline, cognitive impairment, brain damage on neuroimaging and incident dementia in several population-based cohort studies, and has been suggested as a (surrogate) outcome measure in intervention trials.^7–14^ In the last decade, research has identified additional modifiable factors that could be included in dementia risk score and used as behavioral targets for preventive interventions. Based on an umbrella review and Delphi expert study, three additional factors were recently identified and included in an updated LIBRA score (LIBRA2), now also including hearing impairment, social contact, and sleep.^15^

The objective of the present study is to compare the performance of the original and updated LIBRA score for predicting the risk of incident dementia in two large, longitudinal population-based aging studies.

## METHODS

### Study design and population

#### The English Longitudinal Study of Ageing (ELSA)

**Fig. 1:**
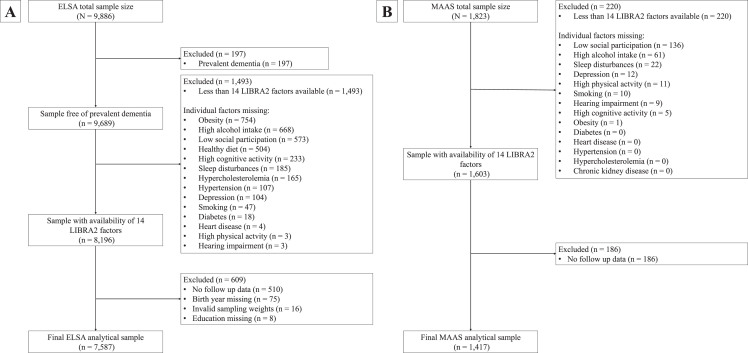
Flowcharts of the study sample selection in ELSA (A) and MAAS (B). ELSA, English Longitudinal Study of Ageing; LIBRA, LIfestyle for BRAin health; LIBRA2, updated LIfestyle for BRAin health; MAAS, Maastricht Aging Study.

ELSA is an ongoing and long-running prospective cohort study designed to uncover determinants of health among the English population aged 50 years and older.^16^ The study started in 2002 and at every wave of the study, separated by two-year intervals, participants engaged in computer-assisted face-to-face interviews and a study questionnaire. Some study waves were supplemented with nurse visits to obtain objective measurements of particular biomarkers. Detailed procedures are described elsewhere.^16^ For the present study, we used Wave 4 (2008/2009; *n* = 9,886) as our baseline wave because it provided the most complete dataset regarding modifiable dementia risk factors. Whenever data on lifestyle factors was missing in Wave 4, we retrieved identical information from either Wave 3 (2006/2007) or Wave 5 (2010/2011). The last assessment was Wave 9 (2018-2019) yielding a maximum follow-up duration of 11 years. Our final study population comprised 7,587 individuals (Fig. 1A).

#### The Maastricht Aging Study (MAAS)

MAAS is a population-based prospective aging study that investigated both successful and pathological aging.^17^ Between 1993 and 1996, a stratified random selection of individuals was made from the Research Network Family Medicine Maastricht, a registration network of family practice in the Province of Limburg, the Netherlands. Stratification was done by gender, 5-year age bands (12 categories), and level of occupational achievement (low, high). The final sample encompassed 1,823 participants aged between 28 and 81 years representative of the general Dutch population. At baseline, the participants underwent extensive neuropsychological and medical testing, in addition to completing lifestyle and general health questionnaires. This cohort was followed for five waves, with the final wave being completed in 2023, yielding a maximum follow-up tine of 25 years. The final study population consisted of 1,417 individuals (Fig. 1B).

### Dementia diagnosis

Dementia diagnosis methods differed between the two cohorts. In ELSA, a diagnosis was based on either (a) a physician’s diagnosis of (Alzheimer’s) dementia reported by the individual or an informant during the computer-assisted personal interview; or (b) the total score on the 16-item Informant Sore Questionnaire on Cognitive Decline in the Elderly (IQCODE).^18^ The IQCODE short-form is an informant-based screening tool to assess changes in an individual’s cognitive abilities over a 2-year period (e.g., ‘Making decisions on everyday matter’). Each of the items is scored on a five-point Likert scale, ranging from 1 (much improved) to 5 (much worse). The cut-off of an average score of 3.38 has proven a useful trade-off between sensitivity (0.79) and specificity (0.82), resulting in an area under the curve of 0.85 for probable dementia diagnosis.^19^

In MAAS, dementia cases were identified based of International Classification of Primary Care codes retrieved from the Research Network Family Medicine Maastricht. In addition, in some instances, a dementia diagnosis was made by an experienced neuropsychiatrist from the Memory Clinic of the Maastricht University Medical Centre+ based on neuropsychological and other clinical data collected during MAAS.

### Assessment of LIBRA and LIBRA2 scores

The LIBRA index originally consisted of twelve modifiable risk and protective factors which could be targeted by lifestyle intervention and vascular risk management in primary care. Weights of the different factors were based on risk estimates from previously published meta-analyses.^4^ Subsequently, the weights were standardized and summed up to calculate one’s LIBRA score (Supplementary Table 1). The theoretical range goes from –5.9 to +12.7 where a higher score represents an unhealthier lifestyle and, thus, higher estimated dementia risk. Recently, we updated the LIBRA index with three new factors: hearing impairment, social contact, and sleep, resulting in the LIBRA2 index.^15^ Based on risk estimates reported in recent meta-analyses for both the original twelve and new three factors, we calculated new weights, and determined the newly reweighted composition of LIBRA2.^15^ Additionally, we created rescaled versions of both LIBRA and LIBRA2, both with theoretical ranges from 0 to 100 (Supplementary Table 2). Both cohorts had data for 14 out of 15 LIBRA2 factors (missing in ELSA: chronic kidney disease; missing in MAAS: healthy diet) resulting in differing LIBRA and LIBRA2 ranges in both cohorts. Risk factor status was dichotomized based on established cut-offs and operationalizations of LIBRA factors differed between cohorts based on the availability of objective (e.g., clinical data from nurse visits, audiometry) and subjective (e.g., self-reported diagnosis, questionnaires) measurements (Supplementary Table 3).

### Statistical analyses

We used independent samples *t*-tests and chi-squared tests to analyze baseline differences in risk/protective factors and sociodemographic variables between participants with and without incident dementia. For our main analysis, we used Cox-proportional hazards regression models to examine the association between LIBRA and LIBRA2 (both as a continuous and a categorical variable (using tertiles)) and time to dementia, resulting in hazard-ratios (HRs) and associated 95% confidence intervals (95% CIs). Additionally, we also investigated the association between the new factors that were included in LIBRA2 (hearing impairment, social contact, sleep) and incident dementia. Only individuals with complete data on all available LIBRA2 factors in either dataset were included in our analyses (complete case analyses). We implemented models with age as the time scale, using the birthdate as the model’s origin and the age at Wave 4 (ELSA) or Wave 0 (MAAS) as the model’s entry point. In all analyses, dementia diagnosis was treated as the failure event. Survival time was defined as the period from birth until the onset of dementia, last interview date, or death (whichever came first). By defining survival time this way, age was considered in the time scale for all analyses. We assessed the proportional hazard assumption using Schoenfeld residuals.^20^ Each analysis using a model with LIBRA or LIBRA2 as the sole predictor (model 0) was followed by an analysis adjusting for age, sex and education (model 1). For both cohorts, a sampling weight (baseline cross-sectional weight) was used to back-weight estimates from the analytical sample to the total sample (ELSA) or Research Network Family Medicine Maastricht source population (MAAS) to minimize selection bias. To assess and compare overall fit of the models, we used the Akaike Information Criterion. Subsequently, we calculated the Harrell’s C-index, also known as concordance index, for each model to determine predictive accuracy. This index reflects the probability that the model ranks a randomly selected dementia case higher than an individual who did not develop dementia or who developed dementia after a longer follow-up time.^21^ It is equal to the area under the Receiver Operating Characteristic curve and ranges from 0.5 to 1. To calculate this statistic, we used models with survival times on the x-axis (as opposed to age), as concordance cannot be computed using delayed-entry data.

In sensitivity analyses, we tested LIBRA or LIBRA2 as a predictor for dementia in midlife subpopulations (40–75 years old) for both cohorts. In these same subpopulations, we also investigated the associations between individual new LIBRA2 factors (hearing impairment, social contact, sleep) and dementia. Additionally, we checked the independent effects of re-weighting the factors and adding new factors by creating a modified LIBRA version (mod-LIBRA) that included only original LIBRA factors but with the weights of LIBRA2. All analyses were done in Stata 17 (StataCorp LP, TX), and the level of statistical significance was *p* < 0.05 in two-sided tests.

### Ethical approval

For the ELSA study, study approval was obtained from the National Health Service Multicentre Research and Ethics Committee as well as the University College London Research Ethics Committee (MREC/01/2/91). Written informed consent was provided by all participants. In MAAS, The Ethics Committee of Maastricht University Medical Centre provided study approval, and all participants gave informed consent (METC2019-1151). No additional ethical approval was needed for this secondary analysis.

## RESULTS

### Population characteristics

Figure 1 provides a flowchart for participant selection. During an average follow-up time of 8.3 years (standard deviation (SD), 2.7), 346 participants in ELSA (4.6%) developed dementia (incidence rate = 53.4 (95% CI, 47.9–59.7) per 10,000 person-years). During an average follow-up time of 17.9 years (SD, 8.9), 120 participants in MAAS (8.5%) developed dementia (incidence rate = 52.2 (95% CI, 43.5–63.3) per 10,000 person-years).

The population characteristics for both studies are shown in Table 1. Individuals with incident dementia were on average older, had a lower education level and were more often female (all *p* < 0.05, except sex distribution in MAAS). In both studies, coronary heart disease, hypertension, depression, and hearing impairment were more prevalent among individuals who developed dementia. Additionally, diabetes and low social contact were more common, while high physical activity and high cognitive activity were less common among the incident dementia group in ELSA. In MAAS, individuals with incident dementia more frequently had obesity. Hypercholesterolemia and high alcohol intake were more common among the individuals without incident dementia in ELSA. Lastly, smoking was more common and high cognitive activity was less common among individuals without incident dementia in MAAS.

**Table 1:** Baseline characteristics of ELSA (Wave 4) and MAAS (Wave 0) participants by incident dementia status

	ELSA (*n* = 7, 587)				MAAS (*n* = 1,417)			
Variable	Total	Dementia	No dementia	*p*	Total	Dementia	No dementia	*p*
	(*n* = 7,587)	(*n* = 346)	(*n* = 7,241)		(*n* = 1,417)	(*n* = 120)	(*n* = 1,297)
Demographics
Age, mean (SD)	65.9 (8.9)	74.9 (7.9)	65.4 (8.7)	<0.001	50.4 (15.8)	66.5 (8.1)	48.8 (15.5)	<0.001
Female, *n* (%)	4,189 (55.2)	212 (61.3)	3,977 (54.9)	0.020	680 (48.0)	61 (50.8)	619 (47.7)	0.514
Educational level, *n* (%)				<0.001				<0.001
Low	3,023 (39.9)	191 (55.2)	2,832 (39.1)		478 (33.7)	60 (50.0)	418 (32.2)
Medium	2,066 (27.2)	77 (22.3)	1,989 (27.4)		599 (42.3)	43 (35.8)	556 (42.9)
High	2,498 (33.0)	78 (22.5)	2,420 (33.4)		340 (24.0)	17 (14.2)	323 (24.9)
Lifestyle factors
Diabetes, *n* (%)	762 (10.0)	58 (16.8)	704 (9.7)	<0.001	53 (3.8)	6 (5.0)	47 (3.6)	0.447
Heart disease, *n* (%)	767 (10.1)	70 (20.2)	697 (9.6)	<0.001	148 (10.4)	22 (18.3)	126 (9.7)	0.003
^a^High physical activity, *n* (%)	5,565 (73.4)	181 (52.3)	5,384 (74.4)	<0.001	874 (61.7)	72 (60.0)	802 (61.8)	0.692
Smoking, *n* (%)	975 (12.9)	33 (9.5)	945 (13.0)	0.059	394 (27.8)	18 (15.0)	376 (29.0)	0.001
Hypertension, *n* (%)	2,528 (33.3)	156 (45.1)	2,372 (32.8)	<0.001	403 (28.4)	60 (50.0)	343 (26.5)	<0.001
Hypercholesterolemia, *n* (%)	3,879 (51.1)	130 (37.6)	3,749 (51.8)	<0.001	138 (9.7)	16 (13.3)	122 (9.4)	0.165
^b^High alcohol intake, *n* (%)	823 (10.9)	22 (6.4)	801 (11.1)	0.006	545 (38.5)	42 (35.0)	503 (38.8)	0.415
Depression, *n* (%)	1,504 (19.8)	107 (30.6)	1,398 (19.3)	<0.001	330 (23.3)	37 (30.8)	293 (22.6)	0.041
High cognitive activity, *n* (%)	4,120 (54.3)	95 (27.5)	4,025 (55.6)	<0.001	451 (31.8)	63 (52.5)	388 (29.9)	<0.001
Obesity, *n* (%)	2,483 (32.8)	106 (30.6)	2,377 (32.8)	0.396	246 (17.4)	30 (25.0)	216 (16.7)	0.021
Healthy diet, *n* (%)	4,449 (58.6)	188 (54.3)	4,261 (58.9)	0.096	NA	NA	NA	NA
Chronic kidney disease, *n* (%)	NA	NA	NA	NA	52 (3.7)	6 (5.0)	46 (3.6)	0.418
Hearing impairment, *n* (%)	1,451 (19.1)	116 (33.5)	1,335 (18.4)	<0.001	383 (27.0)	66 (55.0)	317 (24.4)	<0.001
Low social contact, *n* (%)	4,506 (59.4)	234 (67.6)	4,272 (59.0)	0.001	617 (43.5)	51 (42.5)	566 (43.7)	0.810
Sleep disturbances, *n* (%)	1,623 (21.4)	68 (19.7)	1,555 (21.5)	0.419	433 (30.6)	42 (35.0)	391 (30.2)	0.269
LIBRA score, mean (SD)	–0.4 (3.2)	1.2 (3.0)	–0.5 (3.2)	<0.001	0.7 (2.3)	0.7 (2.2)	0.6 (2.5)	0.643
LIBRA2 score, mean (SD)	2.4 (4.7)	4.6 (4.8)	2.3 (4.7)	<0.001	3.2 (3.8)	4.0 (3.9)	3.1 (3.8)	0.013
Res-LIBRA score, mean (SD)	29.7 (17.1)	38.3 (16.0)	29.3 (17.1)	<0.001	26.7 (12.2)	26.1 (13.6)	26.7 (12.1)	0.609
Res-LIBRA2 score, mean (SD)	26.6 (14.8)	33.6 (15.0)	26.3 (14.7)	<0.001	25.4 (11.9)	28.0 (12.3)	25.1 (11.7)	0.011

Data on chronic kidney disease and healthy diet were missing from ELSA and MAAS, respectively. An overview of how all factor were operationalized can be found in Supplementary Table 3. ^a^The risk factor physical inactivity (as originally included in LIBRA) was converted into a protective factor defined as high physical activity. ^b^The protective factor low-to-moderate alcohol consumption (as originally defined in LIBRA) was converted into a risk factor defined as high alcohol intake. ELSA, English Longitudinal Study of Ageing; LIBRA, LIfestyle for BRAin health; LIBRA2, updated LIfestyle for BRAin health; MAAS, Maastricht Aging Study; Res-LIBRA, rescaled LIfestyle for BRAin health; RES-LIBRA2, rescaled and updated LIfestyle for BRAin health.

In ELSA, LIBRA scores were significantly higher in individuals who developed dementia (mean LIBRA score (SD): 1.2 (3.0)) compared to individuals without incident dementia (mean LIBRA score (SD): –0.4 (3.2)). Consistently, LIBRA2 scores were also significantly higher in people who developed dementia (mean LIBRA2 score (SD) 4.6 (4.8)) compared to individuals who did not develop dementia (mean LIBRA2 score (SD): 2.3 (4.7)). In MAAS, LIBRA2 scores, but not LIBRA scores, were significantly higher among the incident dementia group (mean LIBRA2 score (SD): 4.0 (3.9)) compared to individuals without incident dementia (mean LIBRA2 score (SD): 3.1 (3.8)). Baseline differences between diagnostic groups for the midlife subpopulations (40–75 years) can be found in Supplementary Table 4.

### Original and rescaled LIBRA and LIBRA2 and incident dementia

**Fig. 2:**
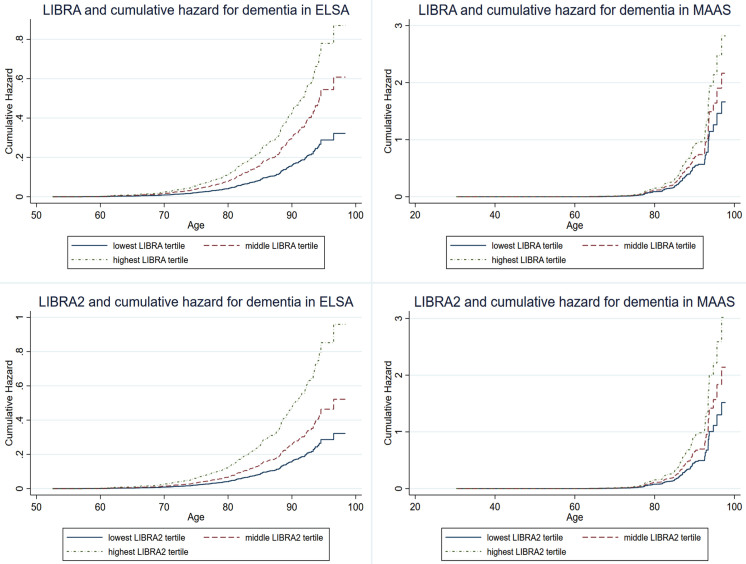
LIBRA and LIBRA2 score tertiles and cumulative hazard of dementia in ELSA and MAAS. ELSA, English Longitudinal Study of Ageing; LIBRA, LIfestyle for BRAin health; LIBRA2, updated LIfestyle for BRAin health; MAAS, Maastricht Aging Study.

Table 2 and Fig. 2 show the performance of the regular and rescaled LIBRA and LIBRA2 scores in predicting dementia. Higher LIBRA scores were associated with increased risk of dementia in ELSA (hazard ratio (HR) = 1.13; 95% confidence interval (CI), 1.09–1.16; C-statistic: 0.68), but not in MAAS (HR = 1.08; 95% CI, 0.99–1.16; C-statistic = 0.52). This association remained significant in ELSA after adjustment for sex and education (HR = 1.13; 95% CI, 1.09–1.17; C-statistic = 0.83) and remained non-significant in MAAS (HR = 1.07; 95% CI, 0.98–1.16; C-statistic = 0.93). Higher LIBRA2 scores were associated with increased dementia risk in ELSA (HR = 1.08; 95% CI, 1.06–1.11; C-statistic = 0.67), as well as in MAAS (HR = 1.06; 95% CI, 1.02–1.11; C-statistic = 0.62). These results remained significant after adjustment for education and sex in both cohorts. Rescaling to a 0–100 scale yielded similar results for LIBRA and LIBRA2 in both studies, with altered, more comparable hazard ratios between both LIBRA versions (Table 2). When LIBRA scores were used as categorical variables (tertiles), the middle and highest LIBRA tertiles predicted higher risk of dementia compared with the lowest tertile in both ELSA (respectively, HR_T2_ = 1.89; 95% CI, 1.35–1.96 and HR_T3_ = 2.70; 95% CI, 1.96–3.72) and MAAS (respectively, HR_T2_ = 1.68; 95% CI, 1.03–2.74 and HR_T3_ = 1.69; 95% CI, 1.10–2.60). Similarly, individuals in the middle and highest LIBRA2 tertiles had higher dementia risk in ELSA (respectively, HR_T2_ = 1.62; 95% CI, 1.16–2.27 and HR_T3_ = 2.98; 95% CI, 2.18–4.07). In MAAS only individuals in the highest tertile had a higher dementia risk (HR_T2_ = 1.56; 95% CI, 0.93–2.62 and HR_T3_ = 2.02; 95% CI, 1.27–3.22).

**Table 2:** Performance of the (rescaled) LIBRA and LIBRA2 score in predicting incident dementia in ELSA and MAAS (total study samples)

		ELSA (*n* = 7,587)				MAAS (*n* = 1,417)			
		HR	95% CI	AIC	Harrell’s C (SE)	HR	95% CI	AIC	Harrell’s C (SE)
Model 0	LIBRA	1.13^*^	1.09–1.16	4486	0.68 (0.013)	1.08	0.99–1.16	1305	0.52 (0.030)
	LIBRA2	1.08^*^	1.06–1.11	4482	0.67 (0.014)	1.06^*^	1.01–1.12	1302	0.62 (0.027)
	Res-LIBRA	1.023^*^	1.016–1.029	4486	0.68 (0.013)	1.014	0.999–1.029	1305	0.52 (0.030)
	Res-LIBRA2	1.026^*^	1.019–1.033	4482	0.67 (0.014)	1.019^*^	1.004–1.035	1302	0.62 (0.027)
		HR	95% CI	AIC	Harrell’s C	HR	95% CI	AIC	Harrell’s C
Model 1	LIBRA	1.13^*^	1.09–1.17	4489	0.83 (0.010)	1.07	0.98–1.16	1306	0.93 (0.007)
	LIBRA2	1.08^*^	1.06–1.11	4486	0.83 (0.010)	1.06^*^	1.01–1.11	1303	0.93 (0.007)
	Res-LIBRA	1.022^*^	1.016–1.029	4489	0.83 (0.010)	1.012	0.997–1.028	1306	0.93 (0.007)
	Res-LIBRA2	1.025^*^	1.017–1.032	4486	0.83 (0.010)	1.018^*^	1.002–1.034	1303	0.93 (0.007)

The hazard ratios are based on Cox proportional hazards regression models with age as the time scale and birthdate as origin. The Harrell’s C concordance indices are based on models with survival time as the time scale. Model 0 is the crude model. Model 1 is adjusted for age, sex and education. AIC, Akaike information criterion; CI, confidence interval; ELSA, English Longitudinal Study of Ageing; HR, hazard ratio; LIBRA, LIfestyle for BRAin health; LIBRA2, updated LIfestyle for BRAin health; MAAS, Maastricht Aging Study; Res-LIBRA, rescaled LIfestyle for BRAin health; RES-LIBRA2, rescaled and updated LIfestyle for BRAin health. ^*^Values are statistically significant (*p* < 0.05).

**Table 3:** Performance of the individual new LIBRA2 factors in predicting incident dementia in ELSA and MAAS (total study samples)

		ELSA (*n* = 7,587)		MAAS (*n* = 1,417)	
		HR	95% CI	HR	95% CI
Model 0	Hearing impairment	1.62^*^	1.28–2.06	1.01	0.69–1.47
	Low social contact	1.74^*^	1.38–2.20	1.20	0.83–1.74
	Sleep disturbances	1.15	0.87–1.52	1.10	0.74–1.63
		HR	95% CI	HR	95% CI
Model 1	Hearing impairment	1.46^*^	1.15–1.87	1.01	0.69–1.48
	Low social contact	1.41^*^	1.10–1.81	1.19	0.82–1.73
	Sleep disturbances	0.95	0.72–1.27	1.04	0.69–1.54

The hazard ratios are based on Cox proportional hazards regression models with age as the time scale and birthdate as origin. Model 0 is the crude model. Model 1 is adjusted for LIBRA score. CI, confidence interval; ELSA, English Longitudinal Study of Ageing; HR, hazard ratio; LIBRA, LIfestyle for BRAin health; MAAS, Maastricht Aging Study. ^*^Values are statistically significant (*p* < 0.05).

In summary, the overall performance and predictive accuracy were better for LIBRA2 (C-statistic = 0.62) as opposed to LIBRA (C-statistic = 0.52) in MAAS. In ELSA, overall performance was better for LIBRA2, with a similar predictive accuracy for LIBRA (C-statistic = 0.68) than LIBRA2 (C-statistic = 0.67).

### The unique contribution of new LIBRA2 factors

Table 3 presents multiple survival analyses assessing the individual effects of hearing impairment, low social contact, and sleep disturbances on incident dementia. The unadjusted models (model 0) show that hearing impairment (HR = 1.62; 95% CI, 1.28–2.06) and low social contact (HR = 1.74; 95% CI, 1.38–2.20) were significant predictors of incident dementia in ELSA, but not sleep disturbances (HR = 1.15; 95% CI, 0.87–1.52). Albeit being attenuated, hearing impairment (HR = 1.46; 95% CI, 1.15–1.87) and low social contact (HR = 1.41; 95% CI, 1.10–1.81) remained significant predictors of incident dementia after adjustment for LIBRA scores (LIBRA without the factor of interest) in ELSA. In MAAS, none of these additional factors was significantly associated with dementia risk.

### Sensitivity analyses

As the LIBRA index was originally developed for the midlife population (40–75 years old), a sensitivity analysis using these subpopulations in both studies was conducted to test the robustness of our findings. A one-point increase in the LIBRA index was associated with an adjusted HR for dementia of 1.17 (95% CI, 1.12–1.23) and 1.06 (95% CI, 0.96–1.16) in ELSA and MAAS respectively. For LIBRA2, these adjusted HRs were 1.13 (95% CI, 1.09–1.17) and 1.06 (95% CI, 1.00–1.12; Supplementary Table 5). As such, the HRs for a one-point increase in either LIBRA or LIBRA2 were slightly higher in the midlife subpopulation of ELSA as opposed to the total study population, but not in MAAS.

For the individual new LIBRA2 factors, we similarly found stronger associations in the ELSA midlife subpopulation compared to the total population for hearing impairment (HR = 2.16; 95% CI, 1.55–3.01), low social contact (HR = 1.80; 95% CI, 1.29–2.50), and a higher non-significant HR for sleep disturbances (HR = 1.37, 95% CI, 0.93–2.01). However, after adjusting for LIBRA score, only hearing impairment remained significantly associated with dementia risk (HR = 1.95; 95% CI, 1.40–2.73). No significant associations were found between the three individual factors and dementia in the MAAS midlife subpopulation (Supplementary Table 6).

To distinguish between the effects of changing the weights of the LIBRA factors and the addition of three new factors to LIBRA2, we calculated a modified LIBRA (mod-LIBRA) including only the original LIBRA factors, but with the weights from LIBRA2. In ELSA, reweighting reduced the C-statistic from 0.68 to 0.65 and resulted in worse overall performance. The subsequent addition of new factors increased the C-statistic to 0.67 and led to a substantial improvement in model performance. In MAAS, both reweighting and factor addition did improve model performance. The former increased the C-statistic from 0.52 to 0.55 and the latter resulted in a further increase to 0.62. These analyses suggest that reweighting as well as factor addition both had their unique contributions to model performance. A full comparison between LIBRA, mod-LIBRA, and LIBRA2 is presented in Supplementary Table 7.

## DISCUSSION

In this study, we found that an unhealthier lifestyle was associated with higher risk of developing dementia in two independent longitudinal studies. This was found for both the original LIBRA index, and the updated LIBRA2 index, with similar risk estimates. In MAAS, LIBRA2 had better overall performance and predictive accuracy compared to the original LIBRA index, but not in ELSA. Both indices remained significant predictors for dementia when adjusted for age, sex, and formal education, with the exclusion of LIBRA in MAAS. Additionally, we found in ELSA that hearing impairment and social contact were still significant predictors for dementia, even after adjusting for other LIBRA factors, highlighting their added value to LIBRA2.

The LIBRA and LIBRA2 index target modifiable factors in primary care. They exclude factors (such as age, sex, years of formal education, genetics) which are very predictive of dementia and therefore commonly included in dementia risk scores such as the Cardiovascular Risk Factors, Aging, and Incidence of Dementia (CAIDE) score or the Australian National University-Alzheimer’s Disease Risk Index (ANU-ADRI) score.^5,6^ Consequently, the predictive accuracy of our models using LIBRA2 are lower (C-statistic = 0.67 in ELSA; C-statistic = 0.62 in MAAS) than these other dementia risk scores.^22^

Sensitivity analyses focusing on participants aged 40–75 showed similar C-statistics, but with substantially lower model fit indices, indicating better overall model performance in the midlife population. Previous studies using LIBRA also reported higher HRs in midlife^8–11^ compared to late-life^9,10^ or oldest-old.^7,10^ Additionally, the effect of multiple individual risk factors for cognitive decline varies with age, with attenuated associations found in the oldest-old (>80 years old).^23^ This particularly highlights the importance of timely improvements in lifestyle for reducing future dementia risk.^24,25^

Considering that LIBRA and LIBRA2 where constructed based on different factors and different risk estimates from meta-analyses, they have differing theoretical ranges, making direct comparison of their risk estimates difficult.^15^ We therefore constructed rescaled versions with a theoretical range of 0–100 for both LIBRA variants. The risk estimates and C-statistic for both rescaled versions were almost identical, suggesting that they perform equally well at predicting future risk of dementia. If the goal of future studies is to compare risk estimates among (modified versions of) dementia risk scores within one cohort, rescaling is advisable. Additionally, rescaling improves the interpretability of the scores compared to using arbitrary scales.

We also created a modified LIBRA version, retaining the original factors but adopting LIBRA2 weights, with differential effects both datasets. Considering that reweighting had a positive effect on model performance in MAAS, but a negative effect on model performance in ELSA, the relationship between the factors included in the LIBRA and LIBRA2 index and dementia is different for both datasets. However, in both datasets, the addition of the three new factors to LIBRA2 did result in improvements in model performance. Additionally, model performance is more comparable between ELSA and MAAS for LIBRA2 as opposed to LIBRA. Therefore, LIBRA2 appears to be a more consistent model for estimating future risk of dementia based on lifestyle factors, although more external validation is warranted. As such, future studies should not only assess the effect of addition of new risk/protective factors to dementia risk scores, but also the weights (based on recent risk estimates from meta-analyses) allocated to each of the factors and the algorithms by which those weights are combined.

Although three new factors were added to LIBRA2, with strong support from both the literature and dementia experts, adding these factors to LIBRA2 did not result in a substantial increase in the accuracy (better C-statistic) of dementia prediction. This means that a considerable portion of dementia risk is not explained by the modifiable factors currently included in LIBRA2. Indeed, others have found that most other existing risk indices’ predictive ability is almost entirely driven by inclusion of age in the model.^26^ Another data-driven approach to developing a dementia risk prediction model highlighted the significant predictive power of age and apolipoprotein E status, with very limited increases in model performance after including more factors into the model.^27^ Additionally, there might be factors for which causal evidence is accumulating, but which are not currently included in LIBRA2 such as pesticide exposure or psychological stress.^15,28,29^

As model performance is comparable between LIBRA2 and the original LIBRA index, our results suggests that either LIBRA or LIBRA2 could be used for the prediction of dementia. However, for public health purposes, LIBRA2 should be preferred as it includes three additional modifiable dementia factors: hearing impairment, social contact, and sleep. Although rather consistent associations have been found between these three factors and dementia risk in observational studies, definite evidence from randomized-controlled trials is still lacking.^30–32^ In fact, a recent randomized-controlled trial showed that hearing aid intervention was associated with a decreased rate of cognitive decline only in individuals with more pre-existing dementia risk factors such as hypertension or diabetes.^33^ This highlights the potential for risk stratification to identify high-risk groups that might benefit the most from lifestyle interventions.

The major strengths of the current study include the use of two independent samples representative of the older British population and general Dutch population, which allowed for comparison of results as well as the large number of LIBRA2 factors available (14 out of 15) in both datasets. Despite some differences in setting (timing, location), data-collection and in the inclusion and operationalization of the LIBRA2 factors between the two cohorts, LIBRA2 seems to perform consistently under different methodological conditions as indicated by similar results observed in both study populations. As ELSA and MAAS are two distinct populations, the consistency of our results also improved the external validity of our study. This supports that LIBRA2 is applicable beyond one specific setting or cohort study, although active efforts should be made to validate LIBRA2 in more diverse settings. Next to using two independent samples, we also made use of sampling weights to ensure that our analytical samples resembled the source populations from which they arose. Additionally, this study used the LIBRA index, which has been extensively validated in scientific literature and which has recently been updated based on the most recent evidence for dementia risk and protective factors.^15^ However, there are also some limitations that need to be addressed. First, our analyses were limited to individuals with complete data on all available LIBRA factors in each dataset. This could have resulted in the selection of generally healthier individuals and, subsequently, attenuation of the “true” association between LIBRA and LIBRA2 and dementia. Indeed individuals in ELSA with less than 14 LIBRA2 factors were on average older (55.7 years vs 50.4 years; *p* < 0.001), had a lower education level (*p* < 0.001), and developed more often dementia (11.8% vs 7.5%; *p* = 0.026) compared to those with complete data on all LIBRA2 factors. In MAAS, individuals with less than 14 LIBRA factors had also a lower educational level (*p* < 0.001). However, there were no differences regarding baseline age (66.5 years vs 66.1 years; *p* = 0.133) and incident dementia status (5.2% vs 4.4%; *p* = 0.175). Selective attrition of unhealthier individuals might have amplified this selection bias as a result of the long follow-up periods, which are inherent to aging-related studies. Second, although the majority of LIBRA2 factors were available in both datasets, there are considerable differences in the operationalizations of these factors. For example, hearing impairment in ELSA was operationalized based on self-reported hearing using a 5-point Likert-scale, while being objectively measured using audiometry in MAAS. These differences in measurements could lead to exposure misclassification, resulting in potentially biased risk estimates. Such misclassification might have been amplified by using self-reported data on certain LIBRA factors, which is prone to recall bias, especially in older adults with emerging cognitive impairment. As a result, differences in the effects sizes for the association between LIBRA, LIBRA2 and the individual new LIBRA2 factors and dementia might have been caused by discrepancies in how the data was collected. Third, individuals from the ELSA covered a narrower and older age range compared to participants from MAAS. This selection was created by using wave 4 as our baseline wave in ELSA. Because of this selection, the applicability of the results found in ELSA are less representative for individuals in early midlife (40–55 years). Fourth, the observed associations might be influenced by other factors, which were not assessed in the current study. For example, our analyses corrected for educational level, but not for other components of socio-economic position (such as (parental) occupation or income level) that could influence both lifestyle choices as well as dementia risk. Fifth, the current analyses are limited to the effect of the sum of the various isolated factors included in LIBRA and LIBRA2 and does not account for potential interactions among these factors. Last, the current analyses do not account for the cumulative effect of an unhealthy lifestyle over time. Longitudinal research that accounts for the dynamic interplay between lifestyle factors throughout the lifespan is essential to inform effective dementia risk reduction strategies.

### Conclusion

Taken together, our results show that LIBRA2 has similar performance as the original LIBRA index, while also including more modifiable factors for dementia. Therefore, LIBRA2, now also including hearing impairment, social contact, and sleep, is a better tool for public health purposes with more entry points for dementia risk reduction. As evidence is accumulating that the effects of modifiable risk factor could be influenced by external factors such as age, genetic background, and socioeconomic position, future studies should employ stratified analyses to create more tailored and effective dementia risk reduction strategies.

## AUTHOR CONTRIBUTIONS

Colin Rosenau (Conceptualization; Formal analysis; Methodology; Project administration; Writing – original draft); Sebastian Köhler (Conceptualization; Funding acquisition; Methodology; Supervision; Writing – review & editing); Martin van Boxtel (Conceptualization; Methodology; Supervision; Writing – review & editing); Huibert Tange (Data curation; Writing – review & editing); Kay Deckers (Conceptualization; Funding acquisition; Methodology; Project administration; Supervision; Writing – review & editing).

## Supplementary Material

Supplementary Material

## Data Availability

ELSA data can be accessed freely through the UK Data Service Portal (https://beta.ukdataservice.ac.uk/datacatalogue/series/series?id=200011). Due to ethical restrictions and privacy regulations, MAAS data is not publicly available. Interested researchers can send a data request to the Maastricht Aging Study management team. Upon reasonable request, the data analysis protocols can be made available.
