# Supplementary material for: Validation of the Updated “LIfestyle for BRAin health” (LIBRA) Index in the English Longitudinal Study of Ageing and Maastricht Aging Study
Source: J Alzheimers Dis. 2024 Oct 8;101(4):1237–48. doi: 10.3233/JAD-240666 (PMC11492037; doi:10.3233/JAD-240666)
Supplement: Supplementary Material [file jad-101-jad240666-s001.pdf]

Supplementary Material

Validation of the Updated "LIfestyle for BRAin health" (LIBRA) Index in the English Longitudinal Study of Ageing and Maastricht Aging Study

Supplementary Table 1. Original weights of the factors included in the LIBRA index

Factor	Relative risk	Natural logarithm of the relative risk	Weight
Low-to-moderate alcohol consumption	0.74	-0.30	-1.0
Coronary heart disease	1.36	0.31	+1.0
Physical inactivity	1.39	0,33	+1.1
Chronic kidney disease	1.39	0.33	+1.1
Diabetes	1.47	0.39	+1.3
Cholesterol	1.54	0.43	+1.4
Smoking	1.59	0.46	+1.5
Midlife obesity	1.60	0.47	+1.6
Midlife hypertension	1.61	0.48	+1.6
Healthy diet	0.60	-0.51	-1.7
Depression	1.85	0.62	+2.1
High cognitive activity	0.38	-0.97	-3.2

The weights for the factors in LIBRA were calculated by taking the natural logarithm of their relative risks. Subsequently those were standardized by taking the lowest absolute value of the natural logarithms as the reference value and dividing each natural logarithm by this value. LIBRA, LIfestyle for BRAin health

Supplementary Table 2. New weights of the factors included in the (rescaled) LIBRA and LIBRA2 index

Factor	Old relative risk	New relative risk	[e]Weight LIBRA	[f]Weight LIBRA2	[g]Weight Res-LIBRA	[h]Weight Res-LIBRA2
[ab]Low-to-moderate alcohol consumption	0.74 [1]	NA	-1.0	NA	+5.4	NA
[bc]High alcohol consumption	NA	1.18 [2]	NA	+1.0	NA	+3.1
Coronary heart disease	1.36 [3]	1.55 [4]	+1.0	+2.6	+5.5	+8.3
[d]Physical inactivity	1.39 [5]	NA	+1.1	NA	+5.9	NA
[ad]High physical activity	NA	0.73 [6]	NA	-1.9	NA	+6.0
Chronic kidney disease	1.39 [7]	1.35 [8]	+1.1	+1.8	+5.9	+5.7
Diabetes	1.47 [9]	1.43 [10]	+1.3	+2.2	+6.9	+6.8
Cholesterol	1.54 [11]	1.54 [11]	+1.4	+2.6	+7.7	+8.2
Smoking	1.59 [12]	1.52 [13]	+1.5	+2.5	+8.3	+7.9
Midlife obesity	1.60 [14]	1.45 [15]	+1.6	+2.2	+8.4	+7.0
Midlife hypertension	1.61 [14]	1.20 [16]	+1.6	+1.1	+8.5	+3.5
[a]Healthy diet	0.60 [17]	0.82 [18]	-1.7	-1.2	+9.1	+3.8
Depression	1.85 [19]	1.98 [20]	+2.1	+4.1	+11.0	+13.0
[a]High cognitive activity	0.38 [11]	0.61 [21]	-3.2	-3.0	+17.3	+9.4
Hearing impairment	NA	1.49 [22]	NA	+2.4	NA	+7.6
Low social contact	NA	1.41 [23]	NA	+2.1	NA	+6.5
Sleep disturbances	NA	1.19 [24]	NA	+1.1	NA	+3.3

[a]For the calculation of the rescaled LIBRA(2) scores, a positive weight was given when the protective factors were absent.
[b]The protective factor low-to-moderate alcohol consumption (as originally defined in LIBRA) was converted into a risk factor defined as high alcohol intake for LIBRA2.
[c]Low-to-moderate alcohol consumption was not simply inverted to identify individuals with high alcohol consumption in ELSA as this would also include individuals that do not consume alcohol. Therefore, a separate variable was calculated.
[d]The risk factor physical inactivity (as originally included in LIBRA) was inverted into a protective factor defined as high physical activity for LIBRA2.
[e]The weights for the factors in LIBRA were calculated by taking the natural logarithms of their old relative risks. Subsequently those were standardized by taking the lowest absolute value of the natural logarithms as the reference value and dividing each natural logarithm by this value.
[f]The weights for the factors in LIBRA2 were calculated by taking the natural logarithms of their new relative risks. Subsequently those were standardized by taking the lowest absolute value of the natural logarithms as the reference value and dividing each natural logarithm by this value.
[g]The weights for the factors in Res-LIBRA were calculated by taking the natural logarithms of their old relative risks. Subsequently, those were standardized by dividing the absolute value of the natural logarithms by the sum of all absolute values of all natural logarithms. Positive weights were allocated whenever the risk factors were present or the protective factors were absent.
[h]The weights for the factors in Res-LIBRA2 were calculated by taking the natural logarithms of their new relative risks. Subsequently, those were standardized by dividing the absolute value of the natural logarithms by the sum of all absolute values of all natural logarithms. Positive weights were allocated whenever the risk factors were present or the protective factors were absent.

ELSA, English Longitudinal Study of Ageing; LIBRA, LIfestyle for BRAin health; LIBRA2, updated LIfestyle for BRAin health; Res-LIBRA, rescaled LIfestyle for BRAin health; RES-LIBRA2, rescaled and updated LIfestyle for BRAin health

REFERENCES (Supplementary Table 2)

1. Anstey KJ, Mack HA and Cherbuin N. Alcohol consumption as a risk factor for dementia and cognitive decline: meta-analysis of prospective studies. *Am J Geriatr Psychiatry* 2009; 17: 542-555.
2. Livingston G, Huntley J, Sommerlad A, et al. Dementia prevention, intervention, and care: 2020 report of the Lancet Commission. *Lancet* 2020; 396: 413-446. 20200730.
3. Kalantarian S, Stern TA, Mansour M, et al. Cognitive impairment associated with atrial fibrillation: a meta-analysis. *Ann Intern Med* 2013; 158: 338-346.
4. Deckers K, Schievink SHJ, Rodriquez MMF, et al. Coronary heart disease and risk for cognitive impairment or dementia: Systematic review and meta-analysis. *PLoS One* 2017; 12: e0184244.
5. Hamer M and Chida Y. Physical activity and risk of neurodegenerative disease: a systematic review of prospective evidence. *Psychol Med* 2009; 39: 3-11.
6. Xu W, Wang HF, Wan Y, et al. Leisure time physical activity and dementia risk: a dose-response meta-analysis of prospective studies. *BMJ Open* 2017; 7: e014706.
7. Etgen T, Chonchol M, Forstl H, et al. Chronic kidney disease and cognitive impairment: a systematic review and meta-analysis. *Am J Nephrol* 2012; 35: 474-482.
8. Deckers K, Camerino I, van Boxtel MP, et al. Dementia risk in renal dysfunction: A systematic review and meta-analysis of prospective studies. *Neurology* 2017; 88: 198-208.
9. Lu FP, Lin KP and Kuo HK. Diabetes and the risk of multi-system aging phenotypes: a systematic review and meta-analysis. *PLoS One* 2009; 4: e4144.
10. Xue M, Xu W, Ou YN, et al. Diabetes mellitus and risks of cognitive impairment and dementia: A systematic review and meta-analysis of 144 prospective studies. *Ageing Res Rev* 2019; 55: 100944.
11. Anstey KJ, Cherbuin N and Herath PM. Development of a new method for assessing global risk of Alzheimer's disease for use in population health approaches to prevention. *Prev Sci* 2013; 14: 411-421.
12. Peters R, Poulter R, Warner J, et al. Smoking, dementia and cognitive decline in the elderly, a systematic review. *BMC Geriatr* 2008; 8: 36.
13. Niu H, Qu Y, Li Z, et al. Smoking and risk for Alzheimer disease: a meta-analysis based on both case-control and cohort study. *J Nerv Ment Dis* 2018; 206: 680-685.
14. Barnes DE and Yaffe K. The projected effect of risk factor reduction on Alzheimer's disease prevalence. *Lancet Neurol* 2011; 10: 819-828.
15. Qu Y, Hu HY, Ou YN, et al. Association of body mass index with risk of cognitive impairment and dementia: A systematic review and meta-analysis of prospective studies. *Neurosci Biobehav Rev* 2020; 115: 189-198.
16. Ou YN, Tan CC, Shen XN, et al. Blood pressure and risks of cognitive impairment and dementia: a systematic review and meta-analysis of 209 prospective studies. *Hypertension* 2020; 76: 217-225.
17. Psaltopoulou T, Sergentanis TN, Panagiotakos DB, et al. Mediterranean diet, stroke, cognitive impairment, and depression: A meta-analysis. *Ann Neurol* 2013; 74: 580-591.
18. Liu YH, Gao X, Na M, et al. Dietary pattern, diet quality, and dementia: a systematic review and meta-analysis of prospective cohort studies. *J Alzheimers Dis* 2020; 78: 151-168.
19. Diniz BS, Butters MA, Albert SM, et al. Late-life depression and risk of vascular dementia and Alzheimer's disease: systematic review and meta-analysis of community-based cohort studies. *Br J Psychiatry* 2013; 202: 329-335.
20. Cherbuin N, Kim S and Anstey KJ. Dementia risk estimates associated with measures of depression: a systematic review and meta-analysis. *BMJ Open* 2015; 5: e008853.
21. Yates LA, Ziser S, Spector A, et al. Cognitive leisure activities and future risk of cognitive impairment and dementia: systematic review and meta-analysis. *Int Psychogeriatr* 2016; 28: 1791-1806.

22. Ford AH, Hankey GJ, Yeap BB, et al. Hearing loss and the risk of dementia in later life. *Maturitas* 2018; 112: 1-11.
23. Kuiper JS, Zuidersma M, Oude Voshaar RC, et al. Social relationships and risk of dementia: A systematic review and meta-analysis of longitudinal cohort studies. *Ageing Res Rev* 2015; 22: 39-57.
24. Shi L, Chen SJ, Ma MY, et al. Sleep disturbances increase the risk of dementia: A systematic review and meta-analysis. *Sleep Med Rev* 2018; 40: 4-16.

Supplementary Table 3. Operationalization of the (rescaled) LIBRA and LIBRA2 index risk and protective factors in ELSA and MAAS

Factor	Operationalization ELSA	Operationalization MAAS	Weight LIBRA	Weight LIBRA 2	Weight Res-LIBRA	Weight Res-LIBRA2
[ab]Low-to-moderate alcohol consumption	(1) Self-reported intake between ≥1 & ≤14 standard units per week, average over last 12 months	(1) Self-reported intake ≤7 standard units per week	-1.0	NA	+5.4	NA
[c]High alcohol consumption	(1) [b]Self-reported intake >14 standard units per week	(1) Inverse of low-to-moderate alcohol consumption	NA	+1.0	NA	+3.1
Coronary heart disease	(1) Self –reported diagnosis of angina or MI	(1) Self-reported presence of cardiovascular disease<br>(2) Self-reported diagnosis of cardiac rhythm disorders, chest pain/angina pectoris, heart attack, heart insufficiency	+1.0	+2.6	+5.5	+8.3
[d]Physical inactivity	(1) Self-reported *sedentary* or *low* physical activity level on a 4-point scale	(1) Self-reported hours up and about, divided in tertiles (lowest tertile)<br>(2) Self-reported hours spent weekly on sports ≥2 hours	+1.1	NA	+5.9	NA
[ad]High physical activity	(1) Inverse of the risk factor *physical inactivity*	(1) Inverse of the risk factor *physical inactivity*	NA	-1.9	NA	+6.0
Chronic kidney disease	Missing	(1) Self-reported diagnosis of kidney disease	+1.1	+1.8	+5.9	+5.7
Diabetes	(1) HbA1c ≥ 6.5%, following WHO guidelines [25]<br>(2) Self-reported diagnosis of diabetes	(1) Self-reported presence of diabetes<br>(2) Self-reported diagnosis of diabetes<br>(3) Diabetes medication use	+1.3	+2.2	+6.9	+6.8
Cholesterol	(1) TC ≥5.0mmol/l and LDL ≥3.0mmol/l, following NHS guidelines [26]<br>(2) Self-reported diagnosis of hypercholesterolemia	(1) Self-reported presence of hypercholesterolemia or cholesterol medication use	+1.4	+2.6	+7.7	+8.2
Smoking	(1) Self-reported smoking	(1) Self-reported smoking	+1.5	+2.5	+8.3	+7.9
Midlife obesity	(1) BMI ≥30<br>(2) Waist circumference >102 cm (men)	(1) BMI ≥30<br>(2) Waist circumference >102 cm	+1.6	+2.2	+8.4	+7.0

	or >88 cm (women) (3) waist-to-hip-ratio >0.9 (men) or >0.85 (women), following WHO guidelines [27]	(men) or >88 cm (women), following WHO guidelines [27]				
Midlife hypertension	(1) Mean SBP ≥140 mmHg or mean DBP ≥90 mmHg, following WHO guidelines [28] (2) Self-reported diagnosis of hypertension	(1) Mean SBP ≥140 mmHg or mean DBP ≥90 mmHg, following WHO guidelines [28] (2) Antihypertensive medication use (3) Self-reported hypertension	+1.6	+1.1	+8.5	+3.5
[a]Healthy diet	(1) Self-reported fruit and vegetable consumption ≥5 portions/day	Missing	-1.7	-1.2	+9.1	+3.8
Depression	(1) Total 8-item CES-D score ≥3 [29]	(1) Total SCL-90 depression subscale score, divided in quartiles (highest quartile)	+2.0	+4.1	+11.0	+13.0
[a]High cognitive activity	(1) Self-reported number of intellectual activities undertakes in last 12 months (e.g., reading the newspaper, having a hobby, going on holidays, …), divided by median split (upper half)	(1) Self-reported hours spent weekly on intellectual activities per week (e.g., reading, mind games, …), divided in tertiles (highest tertile)	-3.2	-3.0	+17.3	+9.4
Hearing impairment	(1) Self-reported *poor* or *fair* hearing on a 5-point scale, following a recent ELSA study [30]	(1) Best-ear pure tone audiometry threshold ≥20 dB	NA	+2.4	NA	+7.6
Low social contact	(1) Self-reported number of social memberships (e.g., charitable association, church or other religious group. Political party, …), divided by median split (lower half), following a recent ELSA study [31]	(1) Self-reported hours spent weekly on activities in associations or clubs, divided in tertiles (lowest tertile)	NA	+2.1	NA	+6.5
Sleep disturbances	(1) Self-reported *very bad* or *fairly bad* overall sleep quality on a 4-point scale (2) Self-reported sleep duration ≤4 hours or >10 hours, following a recent ELSA study [32]	(1) Total SCL-90 sleep subscale score, divided in tertiles (highest tertile)	NA	+1.1	NA	+3.3
Theoretical range			-5.9 to +12.6	-6.1 to +25.8	0 to +100	0 to +100
Range ELSA			-5.9 to	-6.1 to	0 to +94.1	0 to +94.3

	+12.6	+24.0		
Range MAAS	-4.2 to +12.6	-4.9 to +25.8	0 to +90.9	0 to +96.2

Data on chronic kidney disease and healthy diet were missing from ELSA and MAAS, respectively.
[a]For the calculation of the rescaled LIBRA and LIBRA2 scores, a positive weight was given when the protective factors were absent.
[b]The protective factor low-to-moderate alcohol consumption (as originally defined in LIBRA) was converted into a risk factor defined as high alcohol intake for LIBRA2.
[c]Low-to-moderate alcohol consumption was not simply inverted to identify individuals with high alcohol consumption in ELSA as this would also include individuals that do not consume alcohol. Therefore, a separate variable was calculated.
[d]The risk factor physical inactivity (as originally included in LIBRA) was converted into a protective factor defined as high physical activity for LIBRA2.
BMI, body mass index; CES-D, Center for Epidemiologic Studies Depression Scale; DBP, diastolic blood pressure; ELSA, English Longitudinal Study of Ageing; LDL, low-density lipoprotein; LIBRA, LIfestyle for BRAin health; LIBRA2, updated LIfestyle for BRAin health; MAAS, Maastricht Aging Study; Res-LIBRA, rescaled LIfestyle for BRAin health; RES-LIBRA2, rescaled and updated LIfestyle for BRAin health; SBP, systolic blood pressure; SCL, Symptom Checklist 90; TC, total cholesterol

REFERENCES (Supplementary Table 3)

25. World Health Organization. *Use of Glycated Haemoglobin (HbA1c) in the Diagnosis of Diabetes Mellitus: Abbreviated Report of a WHO Consultation*. Geneva, 2011.
26. National Health Service. High cholesterol, https://www.nhs.uk/conditions/high-cholesterol/cholesterol-levels/ (accessed 03-22 2024).
27. World Health Organization. *Waist Circumference and Waist-Hip Ratio: Report of a WHO Expert Consultation*, 2008.
28. World Health Organization. 1999 World Health Organization-International Society of Hypertension Guidelines for the Management of Hypertension. Guidelines Subcommittee. *J Hypertens* 1999; 17: 151-183.
29. Turvey CL, Wallace RB and Herzog R. A revised CES-D measure of depressive symptoms and a DSM-based measure of major depressive episodes in the elderly. *Int Psychogeriatr* 1999; 11: 139-148.
30. Davies HR, Cadar D, Herbert A, et al. Hearing Impairment and Incident Dementia: Findings from the English Longitudinal Study of Ageing. *J Am Geriatr Soc* 2017; 65: 2074-2081.
31. Duffner LA, Deckers K, Cadar D, et al. The role of cognitive and social leisure activities in dementia risk: assessing longitudinal associations of modifiable and non-modifiable risk factors. *Epidemiol Psychiatr Sci* 2022; 31: e5.
32. Ma Y, Liang L, Zheng F, et al. Association between sleep duration and cognitive decline. *JAMA Netw Open* 2020; 3: e2013573.

Supplementary Table 4. Baseline characteristics of ELSA (Wave 4) and MAAS (Wave 0) participants by incident dementia status for participants with a baseline age between 40 and 75 years old.

Variable	ELSA (n = 6,307)				MAAS (n = 946)			
	**Total (n = 6,307)**	**Dementia (n = 172)**	**No dementia (n = 6,135)**	***p***	**Total (n = 946)**	**Dementia (n = 103)**	**No dementia (n = 843)**	***p***
Demographics								
Age, mean (SD)	62.9 (6.3)	68.6 (5.2)	62.7 (6.3)	<0.001	57.2 (10.4)	64.7 (7.2)	56.3 (10.4)	<0.001
Female, n (%)	3,447 (54.7)	96 (55.8)	3,355 (54.6)	0.757	454 (48.0)	54 (52.4)	400 (47.5)	0.340
Educational level, n (%)				<0.001				0.034
Low	2,288 (36.3)	86 (50.0)	2,202 (35.9)		387 (40.9)	53 (51.5)	334 (39.6)	
Medium	1,814 (28.8)	44 (25.6)	1,770 (28.9)		367 (38.8)	37 (35.9)	330 (39.2)	
High	2,205 (35.0)	42 (24.4)	2,163 (35.3)		192 (20.3)	13 (12.6)	179 (21.2)	
Lifestyle factors								
Diabetes, n (%)	583 (9.2)	32 (18.6)	551 (9.0)	<0.001	42 (4.4)	5 (4.9)	37 (4.4)	0.829
Heart disease, n (%)	476 (7.6)	30 (17.4)	446 (7.3)	<0.001	126 (13.3)	17 (16.5)	109 (12.9)	0.313
[a]High physical activity, n (%)	4,879 (77.4)	102 (59.3)	4,777 (77.9)	<0.001	584 (61.7)	62 (60.2)	522 (61.9)	0.733
Smoking, n (%)	887 (14.1)	22 (12.8)	865 (14.1)	0.626	249 (26.3)	18 (17.5)	231 (27.4)	0.031
Hypertension, n (%)	1,972 (31.3)	74 (43.0)	1,898 (30.9)	0.001	344 (36.4)	48 (46.6)	296 (35.1)	0.022
Hypercholesterolemia, n (%)	3,347 (53.1)	70 (40.7)	3,277 (53.4)	0.001	127 (13.4)	14 (13.6)	113 (13.4)	0.958
[b]High alcohol intake, n (%)	731 (11.6)	14 (8.1)	717 (11.7)	0.151	368 (38.9)	36 (35.0)	332 (39.4)	0.384
Depression, n (%)	1,192 (18.9)	59 (34.3)	1,113 (18.5)	<0.001	220 (23.3)	35 (34.0)	185 (22.0)	0.006
High cognitive activity, n (%)	3,759 (59.6)	63 (36.6)	3,696 (60.2)	<0.001	354 (37.4)	53 (51.4)	301 (35.7)	0.002
Obesity, n (%)	2,093 (33.2)	53 (30.8)	2,040 (33.3)	0.503	193 (20.4)	26 (25.2)	167 (19.8)	0.197
Healthy diet, n (%)	3,699 (58.7)	94 (54.7)	3,605 (58.8)	0.280	NA	NA	NA	NA
Chronic kidney disease, n (%)	NA	NA	NA	NA	28 (3.0)	3 (2.9)	25 (3.0)	0.976
Hearing impaired, n (%)	1,078 (17.1)	58 (33.8)	1,020 (16.6)	<0.001	334 (35.3)	55 (53.4)	279 (33.1)	<0.001
Low social participation, n (%)	3,724 (59.1)	117 (68.0)	3,607 (58.8)	0.015	384 (40.6)	41 (39.8)	343 (40.6)	0.863
Sleep disturbances, n (%)	1,382 (21.9)	35 (20.4)	1,347 (22.0)	0.615	323 (34.1)	37 (35.9)	286 (33.9)	0.687
LIBRA score, mean (SD)	-0.7 (3.1)	0.9 (3.2)	-0.7 (3.1)	<0.001	0.8 (2.4)	0.7 (2.6)	0.8 (2.3)	0.619
LIBRA2 score, mean (SD)	2.0 (4.6)	4.5 (5.1)	2.0 (4.6)	<0.001	3.5 (3.9)	4.0 (4.0)	3.4 (3.9)	0.137
Res-LIBRA score, mean (SD)	28.2 (17.0)	36.9 (17.1)	28.0 (16.9)	<0.001	27.0 (12.8)	26.4 (13.8)	27.1 (12.7)	0.606
Res-LIBRA2 score, mean (SD)	25.5 (14.6)	33.2 (16.0)	25.3 (14.5)	<0.001	26.3 (12.3)	28.0 (12.5)	26.1 (12.3)	0.130

Data on chronic kidney disease and healthy diet were missing from ELSA and MAAS, respectively. An overview of how all factor were operationalized can be found in Supplementary Table 3.

[a]The risk factor physical inactivity (as originally included in LIBRA) was converted into a protective factor defined as high physical activity.

[b]The protective factor low-to-moderate alcohol consumption (as originally defined in LIBRA) was converted into a risk factor defined as high alcohol intake.

ELSA, English Longitudinal Study of Ageing; LIBRA, LIfestyle for BRAin health; LIBRA2, updated LIfestyle for BRAin health; MAAS. Maastricht Aging Study; Res-LIBRA, rescaled LIfestyle for BRAin health; RES-LIBRA2, rescaled and updated LIfestyle for BRAin health

Supplementary Table 5. Performance of the (rescaled) LIBRA and LIBRA2 score in predicting incident dementia in ELSA and MAAS for participants with a baseline age between 40 and 75 years old.

		ELSA (n = 6,318)				MAAS (n = 940)			
		**HR**	**95% CI**	**AIC**	**Harrell's C (SE)**	**HR**	**95% CI**	**AIC**	**Harrell's C (SE)**
**Model 0**	LIBRA	1.17*	1.12-1.23	2125	0.67 (0.020)	1.07	0.98-1.16	1085	0.52 (0.030)
	LIBRA2	1.13*	1.09-1.16	2112	0.67 (0.021)	1.06*	1.01-1.12	1081	0.61 (0.027)
	Res-LIBRA	1.030*	1.021-1.039	2124	0.67 (0.020)	1.012	0.997-1.029	1085	0.52 (0.030)
	Res-LIBRA2	1.039*	1.029-1.049	2112	0.67 (0.020)	1.020*	1.004-1.037	1081	0.61 (0.027)
		**HR**	**95% CI**	**AIC**	**Harrell's C (SE)**	**HR**	**95% CI**	**AIC**	**Harrell's C (SE)**
**Model 1**	LIBRA	1.17*	1.12-1.23	2128	0.80 (0.016)	1.06	0.96-1.16	1086	0.87 (0.007)
	LIBRA2	1.13*	1.09-1.17	2115	0.80 (0.016)	1.06*	1.00-1.12	1083	0.88 (0.007)
	Res-LIBRA	1.030*	1.021-1.050	2128	0.80 (0.016)	1.010	0.993-1.028	1086	0.87 (0.007)
	Res-LIBRA2	1.039*	1.029-1.050	2115	0.80 (0.016)	1.018*	1.000-1.036	1083	0.88 (0.007)

The hazard ratios are based on Cox proportional hazards regression models with age as the time scale and birthdate as origin. The Harrell's C concordance indices are based on models with survival time as the time scale. Model 0 is the crude model. Model 1 is adjusted for age, sex and education.

AIC, Akaike information criterion; CI, confidence interval; ELSA, English Longitudinal Study of Ageing; HR, hazard ratio; LIBRA, LIfestyle for BRAin health; LIBRA2, updated LIfestyle for BRAin health; MAAS, Maastricht Aging Study; Res-LIBRA, rescaled LIfestyle for BRAin health; RES-LIBRA2, rescaled and updated LIfestyle for BRAin health

*Values are statistically significant ($p < 0.05$)

Supplementary Table 6. Performance of the individual new LIBRA2 factors in predicting incident dementia in ELSA and MAAS for participants with a baseline age between 40 and 75 years old.

		**ELSA (n = 6,318)**		**MAAS (n = 940)**	
		**HR**	**95% CI**	**HR**	**95% CI**
**Model 0**	Hearing impairment	2.16*	1.55-3.01	1.09	0.74-1.63
	Low social contact	1.80*	1.29-2.50	1.29	0.86-1.95
	Sleep disturbances	1.37	0.93-2.01	1.10	0.72-1.67
		**HR**	**95% CI**	**HR**	**95% CI**
**Model 1**	Hearing impairment	1.95*	1.40-2.73	1.10	0.74-1.63
	Low social contact	1.30	0.91-1.84	1.26	0.83-1.92
	Sleep disturbances	1.04	0.70-1.56	1.04	0.67-1.61

The hazard ratios are based on Cox proportional hazards regression models with age as the time scale and birthdate as origin. Model 0 is the crude model. Model 1 is adjusted for baseline LIBRA score.

CI, confidence interval; ELSA, English Longitudinal Study of Ageing; HR, hazard ratio; LIBRA, LIfestyle for BRAin health; MAAS, Maastricht Aging Study

*Values are statistically significant ($p < 0.05$)

Supplementary Table 7. Effect of reweighing and factor addition on model performance

		ELSA (n = 7,587)				MAAS (n = 1,417)			
		**HR**	**95% CI**	**AIC**	**Harrell's C (SE)**	**HR**	**95% CI**	**AIC**	**Harrell's C (SE)**
**Model 0**	LIBRA	1.13*	1.09-1.17	4485	0.67 (0.013)	1.07	0.99-1.16	1305	0.52 (0.030)
	Mod-LIBRA	1.09*	1.06-1.12	4495	0.65 (0.014)	1.07*	1.01-1.13	1302	0.55 (0.028)
	LIBRA2	1.08*	1.06-1.11	4482	0.67 (0.014)	1.06*	1.01-1.12	1302	0..62 (0.027)
		**HR**	**95% CI**	**AIC**	**Harrell's C (SE)**	**HR**	**95% CI**	**AIC**	**Harrell's C (SE)**
**Model 1**	LIBRA	1.13*	1.09-1.17	4489	0.80 (0.010)	1.07	0.98-1.16	1306	0.93 (0.007)
	Mod-LIBRA	1.08*	1.05-1.11	4498	0.79 (0.010)	1.07*	1.01-1.13	1302	0.93 (0.007)
	LIBRA2	1.08*	1.06-1.11	4486	0.80 (0.010)	1.06*	1.01-1.11	1303	0.93 (0.007)

The hazard ratios are based on Cox proportional hazards regression models with age as the time scale and birthdate as origin. The Harrell's C concordance indices are based on models with survival time as the time scale. Model 0 is the crude model. Model 1 is adjusted for age, sex and education. The modified version of LIBRA (mod-LIBRA) was calculated as LIBRA2 minus the weights for the three newly added LIBRA2 factors (hearing impairment, social contact, sleep).

AIC, Akaike information criterion; CI, confidence interval; ELSA, English Longitudinal Study of Ageing; HR, hazard ratio; LIBRA, LIfestyle for BRAin health; LIBRA2, updated LIfestyle for BRAin health; MAAS, Maastricht Aging Study; mod-LIBRA, modified LIBRA

*Values are statistically significant ($p < 0.05$)
